# Arbuscular mycorrhizal fungi symbiosis enhances growth, nutrient uptake, and oil quality in sunflower–pumpkin under intercropping systems

**DOI:** 10.3389/fpls.2025.1598272

**Published:** 2025-06-18

**Authors:** Aisha A. M. Alayafi, Basmah M. Alharbi, Awatif M. Abdulmajeed, Taghreed S. Alnusaire, Ayshah Aysh ALrashidi, Siham M. AL-Balawi, Hanan Khalaf Anazi, Suliman M. S. Alghanem, Omar Mahmoud Al zoubi, Mona H. Soliman

**Affiliations:** ^1^ Biological Sciences Department, Faculty of Science, University of Jeddah, Jeddah, Saudi Arabia; ^2^ Biology Department, Faculty of Science, University of Tabuk, Tabuk, Saudi Arabia; ^3^ Biodiversity Genomics Unit, Faculty of Science, University of Tabuk, Tabuk, Saudi Arabia; ^4^ Biology Department, Faculty of Science, University of Tabuk, Umluj, Tabuk, Saudi Arabia; ^5^ Department of Biology, College of Science, Jouf University, Sakaka, Saudi Arabia; ^6^ Department of Biology, Faculty of Science, University of Hail, Hail, Saudi Arabia; ^7^ Department of Biology, College of Science, Qassim University, Burydah, Saudi Arabia; ^8^ Biology Department, Faculty of Science Yanbu, Taibah University, Yanbu El-Bahr, Saudi Arabia; ^9^ Biology Department, Faculty of Science, Taibah University, Yanbu El-Bahr, Saudi Arabia; ^10^ Botany and Microbiology Department, Faculty of Science, Cairo University, Giza, Egypt

**Keywords:** arbuscular mycorrhiza colonization, cropping systems, *Cucurbita pepo*, *Helianthus annuus*, nutrient uptake, seed oil quality

## Abstract

**Introduction:**

This study evaluated the effects of *Funneliformis mosseae*, an arbuscular mycorrhizal (AM) fungus, on nutrient uptake, biomass production, and oil quality in sunflower (*Helianthus annuus*) and pumpkin (*Cucurbita pepo*) under both sole and intercropping field systems.

**Methods:**

A factorial experimental design was conducted over two growing seasons (2023 and 2024), involving three cropping systems: sunflower monoculture, pumpkin monoculture, and additive sunflower–pumpkin intercropping. Each system was assessed with and without AM inoculation to determine the interactive effects of mycorrhizal symbiosis and cropping pattern.

**Results:**

AM inoculation significantly improved root colonization, especially in intercropped pumpkins, and enhanced key plant growth parameters, including chlorophyll content, plant height, leaf number, biomass, and reproductive traits. The highest performance was recorded in AM-treated intercropped systems for both crops. Seed weight increased notably under AM inoculation, reflecting improved reproductive development. Analysis of mineral nutrient content revealed substantial improvements in macro- and micronutrient uptake with AM symbiosis. Intercropped AM-treated plants showed enhanced levels of phosphorus, potassium, calcium, zinc, and iron compared to their non-mycorrhizal counterparts. Additionally, AM treatments led to a marked improvement in oil yield and quality. In particular, AM-inoculated sunflower and pumpkin seeds exhibited higher total oil content and a favorable shift in fatty acid composition, characterized by increased oleic acid and reduced linoleic acid concentrations.

**Discussion:**

These findings highlight the synergistic potential of integrating AM fungal inoculation with intercropping practices to enhance crop productivity, nutrient use efficiency, and oilseed quality. The results support the adoption of AM fungi as a sustainable biofertilizer strategy in modern agroecosystems.

## 1 Introduction

Sunflower (*Helianthus annuus* L.) and pumpkin (*Cucurbita pepo* L.) are agriculturally and economically important oilseed crops cultivated worldwide for their nutritional, industrial, and ecological value. Although often studied together in diversified cropping systems, these species differ considerably in their growth habits, agronomic requirements, and oil composition, necessitating a differentiated understanding of their biological and ecological management.

Sunflower is one of the most widely grown oilseed crops globally, with production exceeding 55 million metric tons in 2023 (FAOSTAT, 2024). It is valued for its high-quality edible oil, rich in unsaturated fatty acids, as well as for its potential in biodiesel production, feed, and confectionery uses. Agronomically, sunflower is moderately drought-tolerant and thrives in well-drained soils under full sun, with sensitivity to phosphorus and potassium deficiencies affecting oil yield and quality. The crop’s productivity is influenced by agronomic practices such as crop rotation, nutrient management, and water availability (Albe-Slabi and Kapel, 2024). Current research also emphasizes the need to improve protein valorization and mitigate antinutritional factors in seeds to enhance sunflower’s value in sustainable food systems.

Pumpkin, by contrast, is a warm-season cucurbit with spreading vines and distinct requirements for soil moisture and space. Its seeds are increasingly recognized for their high oil and protein content, as well as bioactive compounds beneficial to human health. Global pumpkin production has increased due to its role in nutrition security and income generation, particularly in smallholder systems (Wargala et al., 2023). Pumpkin requires fertile, well-drained soils and benefits from organic fertilization. Unlike sunflower, pumpkin has a longer growing season and shows high responsiveness to improved nutrient availability.

The selection of sunflower and pumpkin for the intercropping system was based on their complementary morphological and agronomic traits, as well as their individual economic and nutritional importance. Sunflower’s upright growth and deep rooting system contrasts with the sprawling, shallow-rooted nature of pumpkin, allowing for efficient spatial and temporal resource utilization when grown together. This complementary architecture minimizes direct competition and promotes interspecific facilitation, which is a foundational principle in intercropping design (Burgess et al., 2022). Additionally, both crops contribute valuable oilseed products with high market demand and health-promoting properties. While intercropping systems involving sunflower and legumes or cereals have been more extensively studied (Babec et al., 2021; Kinyua et al., 2023; Toker et al., 2024), research involving cucurbits like pumpkin is emerging, showing promise in enhancing yield stability and resource-use efficiency. Therefore, this combination was chosen to explore novel intercropping strategies that align with ecological intensification and sustainable land-use goals.

Enhancing the productivity and quality of these crops is essential to meet the increasing demands for food and industrial raw materials, especially in the face of rising environmental challenges like drought and nutrient depletion (Paladi, 2023). Research emphasizes the importance of sustainable valorization of all seed compounds, including proteins, in sunflowers, and highlights the need for original technological solutions to overcome challenges related to phenolic compounds and antinutritional factors (Albe-Slabi and Kapel, 2024) Additionally, the nutrient background, crop rotation strategies, and fertilization systems significantly impact the yield and quality of sunflowers, showcasing the importance of agricultural practices in optimizing productivity.

Arbuscular mycorrhizal (AM) fungi play a crucial role in enhancing plant performance by improving nutrient acquisition, especially phosphorus (P), potassium (K), zinc (Zn), and iron (Fe), essential for plant physiological processes (Darakeh et al., 2021; Delaeter et al., 2024). This symbiotic relationship is particularly advantageous under stress conditions like drought, where AM fungi contribute to maintaining plant water status, enhancing drought resistance through improved root hydraulic conductivity, and facilitating osmotic adjustment (Wahab et al., 2023b). Additionally, AM fungi have been shown to modulate nutrient stoichiometry, particularly under compound stresses, thereby improving plant resistance to abiotic stress factors like salinity, ultimately aiding in crop yield improvement in saline and arid regions (Ran et al., 2024). The regulatory role of AM fungi in carbon assimilation and nutrient homeostasis under stress conditions provides significant guidance for sustainable agriculture practices and environmental safety (Ran et al., 2024).

AM fungi have been extensively studied for their positive impact on crop growth and productivity. Research by Jia et al. (2023) demonstrated that AM species significantly influenced the eco-physiological characteristics of Imperata cylindrica, enhancing nutrient content and photosynthetic efficiency (Jia et al., 2023). Similarly, Huang et al. (2023) found that AMF inoculation improved wheat growth attributes, chlorophyll content, and nutrient uptake under salt stress conditions (Huang et al., 2023). Moreover, Zhang et al. (2023b) reported that AMF inoculation increased maize biomass, reduced molybdenum transport, and enhanced nutrient uptake, showcasing its potential for phytoremediation (Zhang et al., 2023b). Additionally, the use of AMF as a biofertilizer has been linked to improved drought resistance, nutrient uptake, chlorophyll content, and overall biomass in various crops, as highlighted in the broader literature on AMF benefits (Ezzati Lotfabadi et al., 2022; Khalediyan et al., 2021; Weisany et al., 2016a).

*Funneliformis mosseae*, formerly known as *Glomus mosseae*, is one of the most widely studied and applied species of AMF due to its broad host range and well-documented symbiotic efficiency in cultivated crops. As a member of the Glomeraceae family, *F. mosseae* forms mutualistic associations with the roots of over 80% of terrestrial plant species, facilitating enhanced nutrient uptake—particularly phosphorus and micronutrients such as zinc and iron—through its extensive extraradical hyphal network. In exchange, the host plant provides photosynthetically derived carbon to the fungus, supporting its growth and survival. The presence of *F. mosseae* has been shown to improve plant physiological functions, such as chlorophyll biosynthesis, water use efficiency, and hormonal balance, ultimately contributing to increased biomass and yield (Jia et al., 2023; Mosalman et al., 2024). Its application has proven particularly beneficial in low-input and organic farming systems, where it can reduce reliance on synthetic fertilizers while enhancing crop resilience to abiotic stresses like drought and nutrient-poor soils. Due to its proven compatibility with numerous economically important crops—including cereals, legumes, vegetables, and oilseeds—*F. mosseae* is frequently used in AMF inoculant formulations for sustainable agriculture. Its role in improving not only plant nutrition and growth but also secondary metabolite production and seed quality makes it a promising biological tool for enhancing agroecosystem performance. In this context, its application to crops like sunflower and pumpkin, which differ markedly in their nutrient requirements and growth dynamics, offers a unique opportunity to explore species-specific and system-level responses to AM symbiosis under monocropping and intercropping conditions.

Intercropping has been extensively studied for its benefits in enhancing crop performance and sustainability. Research has shown that intercropping systems not only optimize resource utilization but also reduce pest and disease incidence, improve soil structure, increase microbial diversity, and enhance nutrient availability, creating a conducive environment for AM fungi colonization (Begam et al., 2024; Reddy et al., 2023; Sahoo et al., 2023; Slimani et al., 2023). While the synergistic effects of AM inoculation and intercropping on crop performance have been well-documented, there is a gap in understanding these combined impacts, especially concerning sunflower and pumpkin crops. Exploring the interactions between AM fungi, intercropping, and these specific crops could provide valuable insights into maximizing yield, nutrient uptake, and overall plant health in sustainable agricultural systems.

The primary goal of this study is to investigate the combined effects of AM fungi (*F. mosseae*) inoculation and cropping systems—monocropping and intercropping—on the growth, nutrient content, and seed oil composition of sunflower (*H. annuus*) and pumpkin (*C. pepo*) under field conditions. Specifically, the study aims to assess the extent of root colonization by AM fungi in both crops under different cropping arrangements; evaluate physiological parameters such as chlorophyll content, plant height, and leaf number; determine the effects of AM inoculation on biomass production, including leaf, flower, and seed weight; analyze mineral nutrient concentrations (P, K, Ca, Zn, and Fe) in AM-treated and non-treated plants; and examine the influence of AM fungi on seed oil characteristics, particularly total oil, oleic acid, and linoleic acid content. These objectives are rooted in a growing body of evidence suggesting that AM fungi improve plant nutrition, growth, and stress resilience through enhanced nutrient uptake and metabolic regulation. We hypothesize that AM inoculation will promote higher root colonization, improved physiological traits, greater biomass accumulation, and better oil quality in both crops. Furthermore, we expect that intercropping will act synergistically with AM fungi to optimize nutrient availability and plant productivity, resulting in superior performance compared to monocropping systems. This study aims to provide novel insights into the functional role of AM symbiosis within diversified cropping systems and to inform sustainable agricultural practices that simultaneously improve yield quality and ecological resilience.

## 2 Materials and methods

### 2.1 Experimental design

**Figure 1:**
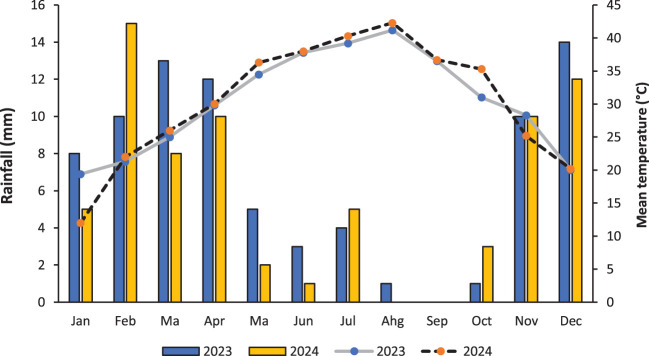
The weather conditions during the period of the experiment.

A field experiment was carried out in the Biology Department, Faculty of Science, Taibah University. **Table 1** shows the physical and chemical properties of the soil. The factorial set of treatments was arranged in a randomized complete block design (RCBD) with three replications. The experimental factors consisted of three cropping systems: (a) sunflower sole cropping (30 plants m^-2^), (b) pumpkin sole cropping (20 plants m^-2^) and (c) additive intercropping of sunflower and pumpkin (30/20 plants m^-2^). Each cropping system was evaluated with (+AM) or without (-AM) arbuscular mycorrhizal colonization to compare the benefits of mycorrhizal symbiosis. The size of each plot was 4 m × 5 m. Hand weeding was carried out after planting. Farm management adhered to organic farming principles, including the use of composted manure as the primary fertilizer, mechanical weed control through hand weeding and mulching, and biological pest management practices; no synthetic fertilizers, pesticides, or herbicides were applied throughout the experiment. The weather conditions during the experimental period are presented in **Figure 1**.

**Table 1:** The soil physicochemical properties.

Year	pH	EC (Ds m^-1^)	OC (%)	N (%)	P (mg kg^-1^)	K (mg kg^-1^)	Sand (%)	Silt (%)	Clay (%)
2023	7.02	0.98	1.05	0.74	65.4	395.2	45	25	30
2024	7.25	1.02	1.12	0.85	75.5	370.4	45	26	29

EC, Electrical Conductivity; OC, Organic Carbon; N, Nitrogen; P, Phosphorus; K, Potassium; pH, potential of Hydrogen.

The AM inoculum used in this study consisted of a blend of colonized root fragments, AM fungal spores, and extraradical hyphae of *Funneliformis mosseae* (strain BEG 119), combined with inert carrier materials to ensure uniform distribution and viability during field application (Jia et al., 2023). The inoculum was produced in a trap culture system using maize as the host plant and was maintained under greenhouse conditions for 16 weeks prior to use. Upon harvesting, the inoculum was carefully mixed with autoclaved sand and a small amount of zeolite to act as inert carriers, which facilitated even mixing and field application. While sand is chemically inert, it also provides physical support and dilution for propagules, helping to distribute the inoculum more uniformly in the root zone at sowing.

Each 30 g portion of the inoculum applied per plot (4 m × 5 m) was standardized based on prior viability assays and was quantified to contain an average of approximately 800–1000 AM fungal spores per gram, along with extensive networks of viable hyphae and colonized root fragments, thereby ensuring a high density of infective propagules. Propagule density and viability were determined using wet sieving and sucrose gradient centrifugation, followed by spore enumeration under a stereomicroscope. While the spore count primarily focused on free spores, the presence of sporocarps (endocarpic spores), which are occasionally formed by some isolates of *F. mosseae*, was specifically evaluated during microscopic examination. However, no sporocarps were observed in the inoculum used, suggesting that the inoculum consisted predominantly of individual spores and extraradical structures. The 30 g application rate was selected based on preliminary field trials and prior recommendations (Wang et al., 2022), demonstrating its effectiveness in achieving robust and consistent root colonization. Inoculum was applied directly into planting holes beneath the seeds at sowing to ensure immediate root contact and optimal establishment of symbiosis.

To ensure that differences in colonization and plant response were attributable to *F. mosseae* inoculation rather than other microbial components, we used a standardized filtered microbial wash in the non-inoculated control. The AM inoculum consisted of a commercial preparation of *F. mosseae* containing spores, colonized root fragments, and hyphal material, which may include native non-AM microorganisms. For control plants, a microbial wash was prepared by filtering the inoculum suspension through Whatman No. 1 filter paper (pore size ~11 µm) to exclude AM propagules while retaining soluble microbial constituents. An equal volume of this filtrate was applied to each control plot to harmonize the microbial background across treatments, in accordance with recommended best practices for AM fungal inoculation studies (Gryndler et al., 2018).

### 2.2 Seed oil extraction and fatty acid composition analysis

To evaluate the effect of AM fungi and cropping systems on seed oil content and composition, mature seeds of sunflower (*Helianthus annuus*) and pumpkin (*Cucurbita pepo*) were harvested at full physiological maturity from each plot. Seeds were air-dried, manually cleaned, and then ground into a fine powder using a laboratory grinder. Approximately 5 g of powdered seed sample was placed in a cellulose extraction thimble and extracted with petroleum ether (boiling point 40–60°C) for 6 hours using a Soxhlet apparatus. After extraction, the solvent was evaporated, and the residual oil was weighed to calculate the percentage of total oil content on a dry weight basis.

Fatty acid composition was analyzed through gas chromatography (GC) following fatty acid methyl ester (FAME) preparation. Briefly, 100 mg of the extracted oil was transesterified with 2 mL of 2% sulfuric acid in methanol by heating at 60°C for 1.5 hours in sealed vials. After cooling, 2 mL of hexane and 1 mL of distilled water were added. The upper hexane layer containing FAMEs was collected and filtered through a 0.22 µm syringe filter. FAMEs were analyzed using a gas chromatograph (Agilent 7890A or equivalent) equipped with a flame ionization detector (FID) and a capillary column (e.g., DB-23, 60 m × 0.25 mm × 0.25 µm). The oven temperature was programmed from 140°C to 220°C at a rate of 4°C/min. Injector and detector temperatures were maintained at 250°C. Identification of oleic acid (C18:1), linoleic acid (C18:2), and other fatty acids was based on comparison with retention times of known FAME standards (Chen and Barak, 1982).

### 2.3 Arbuscular mycorrhizal colonization

Root colonization by arbuscular mycorrhizal (AM) fungi was assessed using a modified version of the staining method initially described by Phillips and Hayman (1970), with procedural adaptations specific to this study (Phillips and Hayman, 1970). Root and rhizosphere soil samples were collected using a 10 mm diameter cylindrical soil corer at a depth of 0–20 cm. After extraction, roots were gently washed under running tap water to remove adhering soil particles.

Washed roots were cut into 1 cm segments and subjected to a clearing step by immersion in 10% (w/v) potassium hydroxide (KOH) at 65 °C for 60 minutes in a water bath. Following KOH treatment, samples were rinsed thoroughly with tap water and then acidified by soaking in 5% lactic acid at room temperature for 12 hours to prepare the tissue for staining.

For staining, a fuscin acid stain was prepared using 0.1 g of fuscin acid dissolved in a solution of 875 mL lactic acid, 63 mL glycerol, and 63 mL distilled water. The root samples were incubated in this staining solution at 70 °C for 30 minutes. Excess stain was removed by destaining the roots in pure lactic acid for 15 minutes.

Ten stained root segments per sample were mounted on microscope slides in a drop of lactic acid and examined under a Nikon YS100 compound microscope (Nikon Corporation, Tokyo, Japan) at magnifications ranging from 100× to 400×.A gridline intersection method (McGonigle et al., 1990) was employed to quantify colonization. An acetate sheet with ten evenly spaced horizontal lines was placed beneath each slide. Each intersection point where a root crossed a line was examined for the presence of AM fungal structures (i.e., arbuscules, vesicles, hyphae). Root colonization was assessed during the peak flowering stage (approximately 10 weeks after sowing) in both 2023 and 2024, corresponding to a period of active root development and established AM symbiosis.

The percentage of root colonization was calculated using the following formula:


Root colonization(%)=(number of colonized rootsegments/number of tested root segments)×100

### 2.4 Chlorophyll content

Chlorophyll content was assessed at the flowering stage using a portable SPAD chlorophyll meter (SPAD-502Plus, Konica Minolta, Japan). Measurements were taken from the uppermost fully expanded leaves of five randomly selected plants per plot to minimize leaf age and positional variability. For each plant, three readings were taken from different parts of the leaf (tip, middle, and base), and the average SPAD value was recorded. These values provide a reliable estimate of relative chlorophyll content and photosynthetic potential. The data were used to compare chlorophyll content among AM-treated and non-treated plants under different cropping systems.

### 2.5 Growth and yield parameters

To evaluate the impact of AM inoculation and cropping systems on vegetative and reproductive growth, key morphological parameters—including plant height, leaf number, leaf weight, flower weight (sunflower), and fruit weight (pumpkin)—were recorded at crop maturity.

Plant height was measured from the base of the stem at soil level to the tip of the tallest shoot using a measuring tape. Measurements were taken from ten randomly selected plants per plot at the physiological maturity stage—approximately 90 days after sowing (DAS) for sunflower and 110 DAS for pumpkin.

Leaf number was counted manually per plant at 75 DAS, when canopy development had reached a stable stage and prior to reproductive senescence. Ten representative plants were sampled per plot, and the number of fully expanded, green leaves was recorded.

Leaf weight was determined by harvesting all fully developed leaves from the same ten sampled plants per plot at 80 DAS. The leaves were washed, air-dried briefly, and weighed using a digital balance to record fresh weight. This parameter served as an indicator of vegetative biomass and photosynthetic potential.

Flower weight (sunflower) was measured at full bloom (~90 DAS) by harvesting flower heads from five randomly selected plants per plot. After removing excess moisture, each flower head was weighed to determine the fresh biomass.

Fruit weight (pumpkin) was assessed at harvest maturity (~110 DAS) by collecting marketable fruits from five randomly selected plants per plot. Fruits were weighed individually, and the average fresh weight per fruit was calculated.

These parameters were selected to comprehensively represent the vegetative growth, source capacity (leaf traits), and reproductive output (flower and fruit development), which are directly influenced by nutrient uptake and AM colonization. The chosen time points align with peak physiological development for each crop, ensuring accurate and meaningful comparisons across treatments.

### 2.6 Plant analysis

The dry ash method (Jones et al., 1991) was used to measure the various elements in the roots and shoots of sunflower (*H. annuus*) and pumpkin (*C. pepo*). The plants were first dried in an oven set at 70°C. Then, 1 g of the dried material was placed in ceramic vessels and gradually heated in the oven to 500°C, resulting in white ash. Once the white ash had cooled to room temperature, we added 20 mL of 1 N HCl to each sample and subjected them to a sand bath for 30 minutes. Each sample was then elutriated using a 100 mL volumetric balloon (Cottenie, 1980). The plant extracts obtained were then used to measure potassium (K) and calcium (Ca) contents using a flame photometer (Model 410, Corning, Halstead, UK) (Williams and Twine, 1960). Iron (Fe) and zinc (Zn) contents were measured using an atomic absorption spectrometer (Shimadzu AA6600, Shimadzu Corporation, Kyoto, Japan) following the method of Jones et al (Jones et al., 1972). Phosphorus (P) was determined by the yellow method using vanadate-molybdate as an indicator (Tandon et al., 1968). The P content was measured at 430 nm using a UV–Vis spectrophotometer (Shimadzu UV3100, Shimadzu Corporation, Kyoto, Japan).

### 2.7 Seed oil content and fatty acid composition

Seed oil content was determined using the Soxhlet extraction method, following AOAC Official Method 920.39 (AOAC (Association of Official Analytical Chemists) and Cunniff, 1990). Mature seeds of sunflower and pumpkin were oven-dried at 60 °C to a constant weight and finely ground using a laboratory mill. Approximately 5.0 g of each ground seed sample was placed in a cellulose thimble and extracted with petroleum ether (boiling range 40–60 °C) for 6 hours in a Soxhlet apparatus. The solvent was then evaporated using a rotary evaporator, and the residual oil was dried and weighed. Oil content was expressed as a percentage of seed dry weight.

Fatty acid composition of the extracted seed oil was analyzed by gas chromatography (GC) after conversion of fatty acids to their corresponding methyl esters (FAMEs). Briefly, 100 mg of the extracted oil was dissolved in 2 mL of hexane, and 2 mL of 0.5 M methanolic KOH was added. The mixture was vortexed and allowed to stand for 10 minutes at room temperature to complete transesterification. The upper hexane layer containing FAMEs was collected and analyzed using a gas chromatograph (Agilent 7890A GC) equipped with a flame ionization detector (FID) and a capillary column (HP-88, 100 m × 0.25 mm × 0.20 µm). The injector and detector temperatures were set at 250 °C and 260 °C, respectively. The oven temperature was programmed to hold at 140 °C for 5 minutes, then increase at 4 °C/min to 240 °C, and hold for 15 minutes. Helium was used as the carrier gas at a constant flow rate. Fatty acids were identified by comparing retention times with known FAME standards and quantified as a percentage of total identified fatty acids. The main components analyzed included linoleic acid (C18:2), oleic acid (C18:1), palmitic acid (C16:0), and stearic acid (C18:0).

### 2.8 Statistical analysis

A variance analysis was conducted using the statistical software package SAS version 9.1 (Statistical Analysis Software (SAS), 2004). The treatment means were compared using orthogonal comparisons. The mean separation was determined using the Generalized Linear Model (GLM) method and the Least Significant Difference (LSD) at a 5% probability level. As the data were normally distributed, transformation was not required.

## 3 Results and discussion

### 3.1 Seed oil composition

The enhancement of total oil and oleic acid content in both sunflower and pumpkin seeds under AM treatments underscores the beneficial influence of AM fungi in modulating seed oil quality. Sunflower seeds under AM intercropping exhibited the highest total oil content (43.04%) and oleic acid (23.18%), whereas non-AM intercropping favored linoleic acid accumulation (61.62%). Similarly, pumpkin seeds showed maximum total oil (47.98%) and oleic acid (27.36%) under AM monocropping, while non-AM monocropping conditions led to the highest linoleic acid content (55.12%) (**Table 2**).

**Table 2:** Seed oil composition of sunflower (*Helianthus annuus*) and pumpkin (*Cucurbita pepo*) seeds under monocropping (Mono) and intercropping (Inter) systems, with (AM) and without (Non-AM) arbuscular mycorrhiza colonization in 2023 and 2024.

Year	Plants	Intercropping	Mycorrhiza	Total oil content (%)	Linoleic acid (%)	Oleic acid (%)	Palmitic acid (%)	Stearic acid (%)
2023	Sunflower seed	Mono	Non-AM	40.56 ± 1.20 d	60.06 ± 1.50 a	20.03 ± 1.00 c	10.42 ± 0.75 ab	5.63 ± 0.50 a
	Sunflower seed	Mono	AM	42.47 ± 1.10 c	58.04 ± 1.25 ab	22.02 ± 1.05 c	11.36 ± 0.80 a	5.58 ± 0.45 a
	Sunflower seed	Inter	Non-AM	39.17 ± 1.25 d	61.62 ± 1.40 a	19.05 ± 1.10 d	10.60 ± 0.85 ab	4.47 ± 0.40 b
	Sunflower seed	Inter	AM	43.04 ± 1.15 c	57.07 ± 1.30 b	23.18 ± 1.00 b	12.52 ± 0.95 a	5.63 ± 0.50 a
	Pumpkin seed	Mono	Non-AM	45.42 ± 1.30 b	55.12 ± 1.40 c	25.68 ± 1.25 ab	8.22 ± 0.60 c	4.75 ± 0.40 b
	Pumpkin seed	Mono	AM	47.98 ± 1.20 a	53.36 ± 1.35 d	27.36 ± 1.15 a	9.02 ± 0.75 b	4.01 ± 0.35 b
	Pumpkin seed	Inter	Non-AM	44.52 ± 1.25 b	56.09 ± 1.50 b	24.48 ± 1.10 b	8.09 ± 0.55 c	3.69 ± 0.35 c
	Pumpkin seed	Inter	AM	46.63 ± 1.20 a	54.75 ± 1.40 d	26.55 ± 1.20 a	9.14 ± 0.60 b	4.08 ± 0.40 b
2024	Sunflower seed	Mono	Non-AM	41.00 ± 1.20 d	61.80 ± 1.55 a	20.50 ± 1.00 c	10.50 ± 0.70 ab	5.50 ± 0.50 a
	Sunflower seed	Mono	AM	43.00 ± 1.15 c	57.50 ± 1.30 ab	22.30 ± 1.10 c	12.50 ± 0.90 a	6.02 ± 0.40 a
	Sunflower seed	Inter	Non-AM	39.80 ± 1.25 d	63.20 ± 1.50 a	19.50 ± 1.20 d	10.80 ± 0.80 ab	4.50 ± 0.40 b
	Sunflower seed	Inter	AM	43.50 ± 1.10 c	56.80 ± 1.40 b	23.50 ± 1.15 b	13.70 ± 0.85 a	6.11 ± 0.45 a
	Pumpkin seed	Mono	Non-AM	46.00 ± 1.25 b	50.80 ± 1.35 c	26.00 ± 1.10 ab	8.50 ± 0.60 c	4.70 ± 0.45 b
	Pumpkin seed	Mono	AM	51.50 ± 1.20 a	48.90 ± 1.30 d	30.80 ± 1.25 a	9.30 ± 0.75 b	4.10 ± 0.40 b
	Pumpkin seed	Inter	Non-AM	44.00 ± 1.30 b	55.90 ± 1.40 b	25.00 ± 1.15 b	8.20 ± 0.55 c	3.70 ± 0.35 c
	Pumpkin seed	Inter	AM	48.20 ± 1.15 a	54.40 ± 1.50 d	29.90 ± 1.20 a	9.50 ± 0.70 b	4.00 ± 0.40 b

Means sharing the same letter are not significantly different at the 5% probability level according to LSD tests.

Data represent the means of three replicates (± SE).

These patterns align with previous studies highlighting that AM fungi enhance the biosynthesis of monounsaturated fatty acids, particularly oleic acid, in oil-rich seeds (Wu et al., 2025; Wang et al., 2022). The observed shift from linoleic to oleic acid in AM-treated seeds may be attributed to the upregulation of desaturase enzymes, especially stearoyl-ACP desaturase and oleate desaturase, which are known to be influenced by symbiotic signaling and improved phosphorus uptake facilitated by AM fungi (Brands and Dörmann, 2022).

Moreover, Wu et al. (2025) reported that co-inoculation of *Rhizophagus intraradices* with beneficial rhizobacteria such as *Burkholderia arboris* enhanced seed oil quality by increasing unsaturated fatty acids (C18:1 and C18:2), supporting the hypothesis that microbial interactions at the rhizosphere level can fine-tune lipid biosynthesis pathways. Our findings reinforce this, indicating that AM symbiosis selectively boosts oleic acid while potentially limiting linoleic acid accumulation, a phenomenon that has also been observed in soybean and flax under similar treatments (Ezzati Lotfabadi et al., 2022)

The greater oleic acid content observed in AM treatments is significant from a nutritional and commercial standpoint, as oleic acid is more oxidatively stable than linoleic acid and contributes to improved shelf-life and health benefits (e.g., cardiovascular protection) of edible oils (Gholinezhad and Darvishzadeh, 2021). These findings suggest that AM inoculation, particularly under intercropping systems, could be strategically leveraged to enhance oil yield and improve fatty acid composition in a sustainable and biologically driven manner (Mosalman et al., 2024). Future research should delve into the gene expression profiles of key enzymes in lipid metabolism in response to AM colonization and explore the synergistic effects of combining AM fungi with specific cropping practices or soil amendments (Figueira-Galán et al., 2023). These approaches may unlock further potential in optimizing oilseed quality through eco-friendly agricultural innovations.

### 3.2 Root colonization

**Figure 2:**
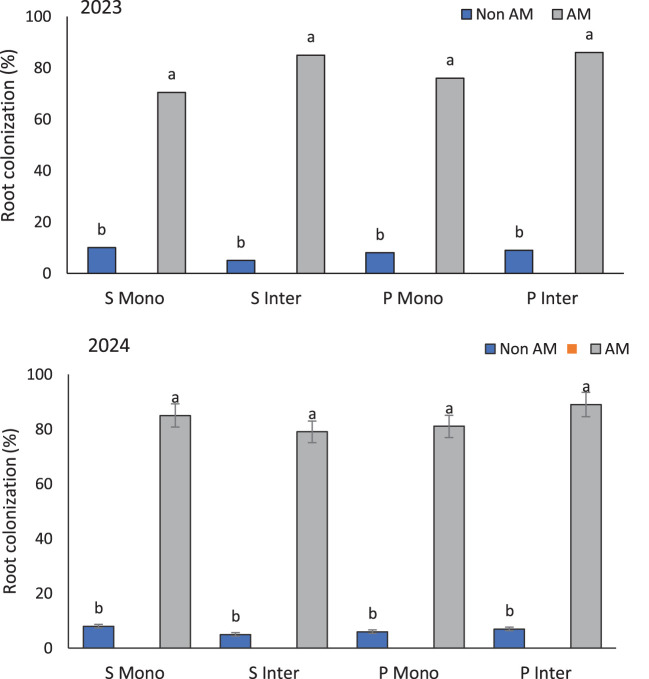
Root colonization percentage of sunflower and pumpkin under mono- and intercropping systems, with and without arbuscular mycorrhizal (AM) inoculation, across the 2023 and 2024 growing seasons. Data are presented separately for each year. Values represent the means of three replicates per treatment. Different letters indicate significant differences among treatments at the 5% probability level according to LSD tests. *S Mono* and *S Inter* refer to sunflower grown in monoculture and intercropping systems, respectively; *P Mono* and *P Inter* refer to pumpkin grown in monoculture and intercropping systems, respectively.

The marked increase in root colonization under AM-inoculated treatments highlights the successful establishment of symbiotic relationships between *F. mosseae* and host crops. The highest root colonization rates, recorded in intercropped AM-treated pumpkins (86% in 2023 and 89% in 2024), suggest that intercropping creates a more favorable environment for the proliferation and establishment of arbuscular mycorrhizal fungi compared to monocropping systems (**Figure 2**). This enhanced colonization may result from improved rhizospheric conditions, including increased microbial diversity, a broader spectrum of root exudates, and enhanced soil structure typically associated with plant diversity (Dlamini et al., 2022). However, it is important to acknowledge that native AM fungi populations present in the experimental soil may have contributed to baseline colonization levels in non-inoculated treatments. While the observed differences between inoculated and non-inoculated plants clearly demonstrate the efficacy of the introduced *F. mosseae*, the role of indigenous AMF communities in supporting plant growth and nutrient acquisition cannot be ruled out. Future studies employing molecular tools or trap culture methods could help differentiate between native and introduced AMF colonization, thereby providing a more nuanced understanding of AMF dynamics under field conditions. This underscores the importance of evaluating both exogenous inoculum performance and native fungal contributions when assessing the potential of AM-based interventions in diverse agroecosystems.

Previous studies support this observation. For instance, Smith and Smith (2011) demonstrated that AM colonization is significantly influenced by host plant species and cropping systems, with intercropping enhancing root colonization due to complementary nutrient uptake patterns and spatial root distribution (Smith and Smith, 2011). Moreover, intercropping systems promote belowground biodiversity, which benefits AM fungi establishment and persistence (Yu et al., 2022). Pumpkin roots showed similarly high colonization rates under monocropping (76% in 2023 and 81% in 2024), indicating that this crop has a high affinity for AM colonization regardless of cropping arrangement. This intrinsic compatibility might be linked to specific root exudates or receptor genes that facilitate AM fungal recognition and infection (Zhang et al., 2024). The findings affirm that root colonization is not solely governed by external cropping conditions but also by the plant’s physiological receptivity. Enhanced root colonization is often associated with improved nutrient absorption, drought tolerance, and resistance to soil pathogens (Rolli et al., 2015). Therefore, the consistently high colonization levels in pumpkins, particularly under intercropping, suggest promising implications for sustainable crop management and resilience under biotic and abiotic stresses.

### 3.3 Chlorophyll content

**Figure 3:**
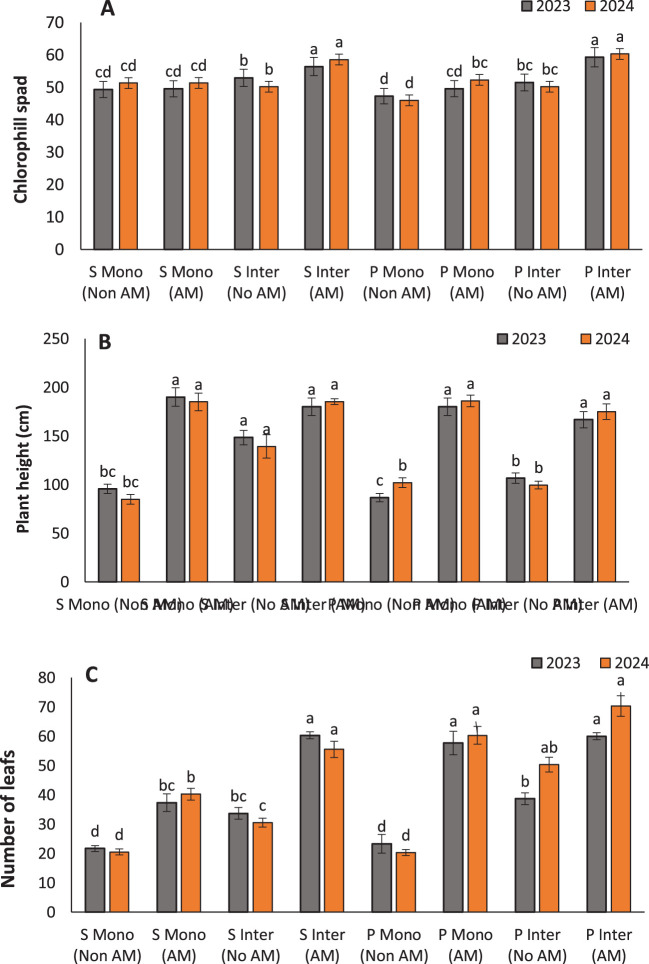
Chlorophyll content **(A)**, plant height **(B)**, and leaf number **(C)** of sunflower (S) and pumpkin (P) under monocropping (Mono) and intercropping (Inter) systems, with (AM) and without arbuscular mycorrhiza (Non AM) colonization in 2023 and 2024. Data represent the means of three replicates. Means sharing the same letter are not significantly different at the 5% probability level according to LSD tests.

Chlorophyll content increased significantly in both sunflowers and pumpkins under AM inoculation, particularly in intercropping systems. The highest chlorophyll levels were recorded in intercropped AM-treated sunflowers (56.43) and pumpkins (59.3), underscoring the synergistic effects of AM fungi and intercropping on enhancing photosynthetic capacity (**Figure 3A**). This improvement can be attributed to better nutrient uptake—especially phosphorus, magnesium, and nitrogen—which are crucial for chlorophyll biosynthesis (Begum et al., 2022; Ullah et al., 2024). Recent work by Slimani et al. (2023) corroborates our findings, showing that AM fungi not only improve the uptake of essential nutrients but also activate physiological pathways involved in chlorophyll synthesis. The mycorrhizal association can induce higher expression of chloroplast-related genes and enhance the photosynthetic efficiency of plants (Boorboori and Lackóová, 2025).

Furthermore, intercropping may enhance these effects by facilitating nutrient sharing and improving soil moisture retention, both of which can further support chlorophyll production (Nasar et al., 2024). Intercropping systems have also been shown to reduce light competition and provide microclimatic benefits, contributing to more favorable conditions for photosynthesis (Maitra et al., 2023). The increased chlorophyll content implies not just improved photosynthesis but also greater energy availability for plant growth and yield. This highlights the strategic value of AM fungi and intercropping in optimizing plant physiological performance under sustainable farming practices.

### 3.4 Plant height, leaf number, leaf weight, flower and fruit weight

**Figure 4:**
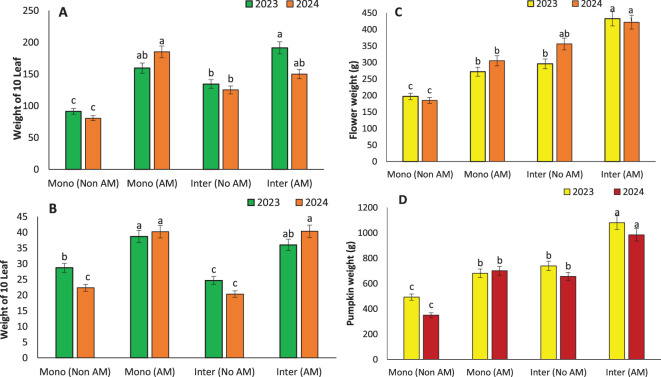
Weight of 10 leaves in sunflower **(A)**, weight of 10 leaves in pumpkin **(B)**, flower weight in sunflower **(C)**, and pumpkin weight **(D)** under monocropping (Mono) and intercropping (Inter) systems, with (AM) and without (Non AM) arbuscular mycorrhiza colonization in 2023 and 2024. Data represent the means of three replicates. Means sharing the same letter are not significantly different at the 5% probability level according to LSD tests.

AM inoculation significantly enhanced vegetative and reproductive growth traits, with the most pronounced effects observed under intercropping systems. The tallest plants were recorded in AM-treated sunflowers (180 cm) and pumpkins (166.67 cm) grown in intercropping systems, indicating that AM symbiosis promotes vertical growth likely due to enhanced nutrient and water uptake (**Figure 3B**). This agrees with earlier studies by Fall et al. (2023), who linked increased plant height to improved phosphorus assimilation and better root architecture induced by AM colonization. Leaf development was similarly stimulated. The highest number of leaves was found in intercropped AM-treated pumpkins (60) (**Figure 3C**), consistent with findings by udwig-Müller and Güther, 2007, who reported that AM fungi can influence hormonal signaling, particularly cytokinins and auxins, to promote leaf proliferation (Ludwig-Müller and Güther, 2007). Enhanced nutrient uptake, especially nitrogen and potassium, also supports greater leaf area development, contributing to increased photosynthetic capacity. Leaf weight followed a similar trend, with intercropped AM-treated pumpkins showing the highest values (191.33 g) (**Figures 4A, B**). This can be interpreted as a proxy for total biomass and is often correlated with plant vigor and health. Fall et al. (2023) reported similar trends in other AM-associated crops, attributing increased biomass to the robust sink-source relationships formed due to enhanced carbon assimilation in mycorrhizal plants.

Reproductive traits also benefited from AM treatment. In sunflowers, the highest flower weight (432.3 g) was recorded under AM intercropping, while in pumpkins, fruit weight peaked under the same conditions (1080.8 g) (**Figures 4C, D**). These gains are crucial, as reproductive biomass is directly linked to yield. The mycorrhizal symbiosis not only improves nutrient translocation to reproductive organs but also enhances assimilate partitioning, ensuring greater allocation of resources to flowers and fruits (Darakeh et al., 2022; Wahab et al., 2023a). The collective improvements in growth and reproductive traits emphasize the holistic benefits of AM fungi, especially under intercropping systems, in fostering plant development and maximizing productivity (Weisany et al., 2016b). These findings advocate for the incorporation of AM fungi into integrated cropping systems for sustainable and yield-efficient agriculture.

### 3.5 Seed weight

**Figure 5:**
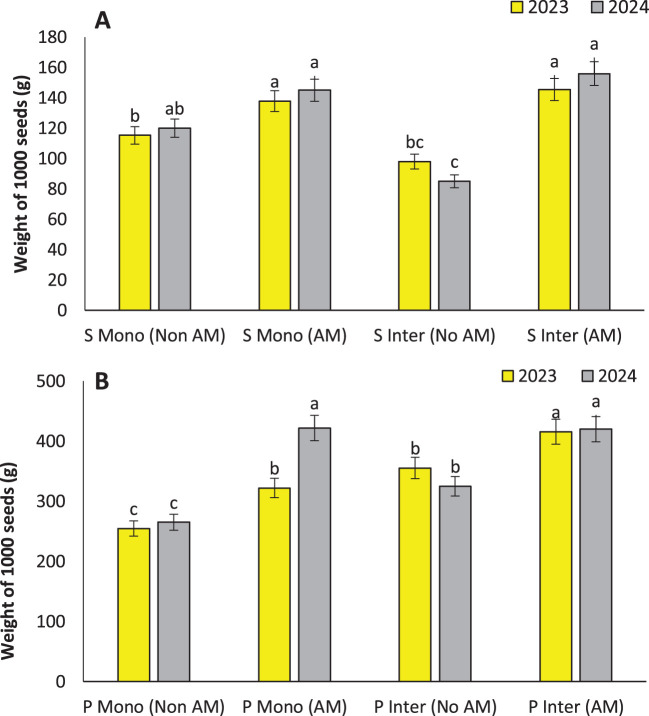
Weight of 1000 seeds of sunflower **(A)** and weight of 1000 seeds of pumpkin **(B)**, under monocropping (Mono) and intercropping (Inter) systems, with (AM) and without (Non AM) arbuscular mycorrhiza colonization in 2023 and 2024. Data represent the means of three replicates. Means sharing the same letter are not significantly different at the 5% probability level according to LSD tests.

The observed increase in seed weight under AM inoculation in both sunflower and pumpkin, particularly under intercropping, demonstrates the central role of AM fungi in enhancing reproductive efficiency and seed filling. These findings align with earlier research where AM colonization significantly increased seed weight and yield in cereal and legume crops due to enhanced nutrient uptake and carbon allocation (Huang et al., 2023). The highest seed weight in intercropped AM-treated pumpkins (415.73 g per 1000 seeds) and sunflowers (145.47 g per 1000 seeds) is indicative of optimized sink-source relationships facilitated by improved nutrient mobilization and hormonal signaling associated with AM colonization (**Figures 5A, B**).

AM-induced improvements in root biomass and soil structure enhance the plant’s capacity to absorb key nutrients such as phosphorus, zinc, and iron—all critical to seed formation (Thangavel et al., 2022). When combined with intercropping, these effects may be synergistic due to increased rhizospheric diversity and nutrient availability (Chamkhi et al., 2022). These results collectively highlight that AM fungi can act as effective biofertilizers in low-input farming systems, particularly when integrated with intercropping strategies, to sustainably increase seed yield and quality across crop types.

### 3.6 Phosphorus

**Figure 6:**
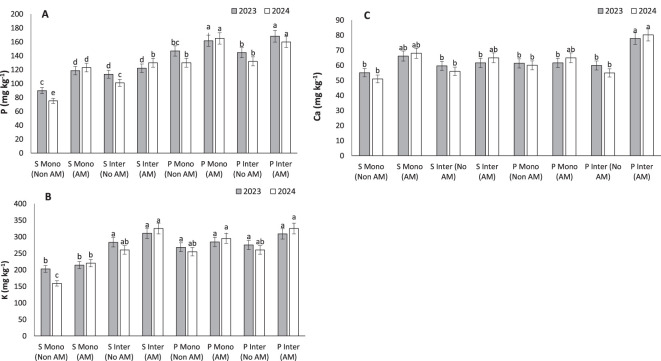
Phosphorous **(A)**, potassium **(B)** and caladium **(C)**, under monocropping (Mono) and intercropping (Inter) systems, with (AM) and without (Non AM) arbuscular mycorrhiza colonization in 2023 and 2024. Data represent the means of three replicates. Means sharing the same letter are not significantly different at the 5% probability level according to LSD tests.

The substantial increase in phosphorus content observed in AM-treated pumpkins (168.14 mg kg⁻¹) under intercropping underscores the mycorrhizal enhancement of P acquisition, a nutrient often limiting in agroecosystems (**Figure 6A**). These results are consistent with findings from Jin et al. (2024), who demonstrated that AM fungi release sugars and phosphatase enzymes that mobilize both inorganic and organic P fractions in soil (Jin et al., 2024). This phosphorus acquisition is primarily mediated by the extraradical hyphae (ERH), which can extend several centimeters beyond the root zone, increasing the effective root-soil interface (Smith and Smith, 2011). Hyphal exudates, including fructose, glucose, and trehalose, further promote phosphatase activity and support microbial populations involved in P cycling (Wang et al., 2023). Zhang et al. (2023b) also reported that AM fungi alleviate nitrogen-induced P limitation in forest soils by solubilizing organic P through ECM-like mechanisms. These processes reflect a dynamic and mutualistic network between AM fungi and rhizospheric microorganisms, suggesting that mycorrhizal strategies could be instrumental in optimizing phosphorus use efficiency in both monoculture and intercropping systems, especially in P-deficient soils.

### 3.7 Potassium

The highest potassium concentrations were recorded in AM-treated intercropped pumpkins (310.48 mg kg⁻¹) and sunflowers (283.4 mg kg⁻¹), indicating that AM fungi can significantly enhance K uptake (**Figure 6B**). Potassium is indispensable for osmoregulation, enzyme activation, and stress tolerance. Wu et al. (2025) confirmed similar trends in legumes and cereals, where AM inoculation increased both P and K levels, directly influencing stomatal regulation and metabolic activities (Wu et al., 2025).

Carrara and Heller (2022) reported that certain AM fungal species, especially Rhizophagus intraradices, selectively enhance uptake of K, Ca, and Mg by increasing transporter gene expression in host roots (Carrara and Heller, 2022). Furthermore, the structure of the intercropping system likely facilitates complementary root interactions, improving nutrient mobility and minimizing intra-species competition (Zhang et al., 2023a). These findings suggest that AM fungi, when combined with intercropping, offer a potent biological solution to enhance potassium nutrition—critical for improving crop tolerance to salinity, drought, and other abiotic stresses.

### 3.8 Calcium

Calcium levels were significantly increased under AM intercropped conditions, with pumpkins reaching 77.82 mg kg⁻¹ (**Figure 6C**). This result aligns with Fu et al. (2023), who observed that AM colonization enhances Ca translocation through improved root morphology and the activity of calcium transporters (Fu et al., 2023).

Calcium serves as a signaling molecule in plants, controlling a range of physiological responses from cell division to stress defense (Tuteja and Mahajan, 2007). Mori and Schroeder (2004) showed that Ca²⁺ signaling underpins defense responses by triggering ROS production and activating stress-related genes (Mori and Schroeder, 2004). Cerella et al. (2010) added that calcium also regulates reproductive development and fertilization by acting as a second messenger in signal transduction pathways (Cerella et al., 2010). In the context of intercropping, enhanced soil biological activity and organic matter may further improve Ca availability and uptake (Ma et al., 2017), suggesting a synergistic interaction between cropping system design and mycorrhizal function.

### 3.9 Zinc

**Figure 7:**
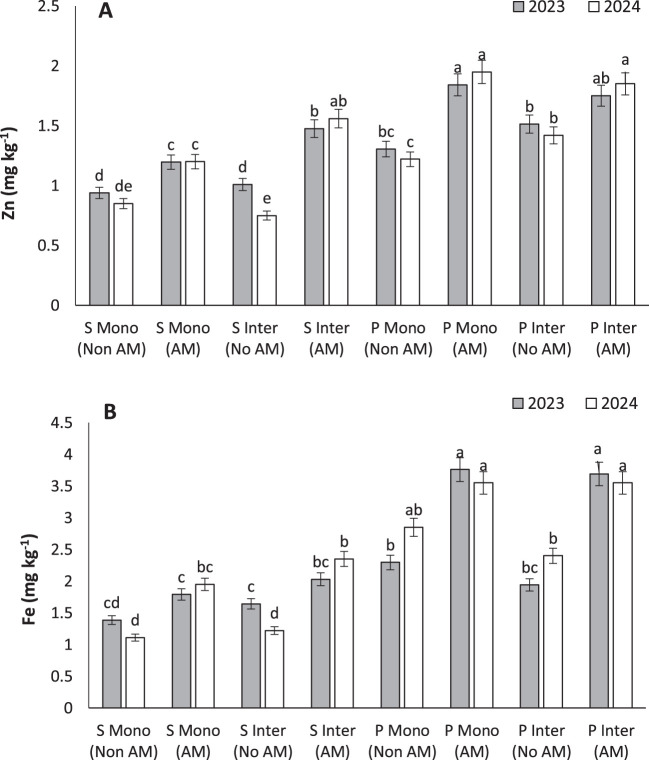
Zinc **(A)** and iron **(B)** under monocropping (Mono) and intercropping (Inter) systems, with (AM) and without (Non AM) arbuscular mycorrhiza colonization in 2023 and 2024. Data represent the means of three replicates. Means sharing the same letter are not significantly different at the 5% probability level according to LSD tests.

AM treatment led to the highest Zn concentrations in pumpkin seeds under both cropping systems (1.84 and 1.75 mg kg⁻¹) (**Figure 7A**). Zinc is essential for enzyme function, protein synthesis, and reproductive development. The current findings are consistent with previous reports by Saboor et al. (2021) and Zhang et al. (2024), which demonstrated that AM fungi substantially improve Zn bioavailability and uptake in multiple plant species, especially under marginal soil conditions (Saboor et al., 2021). Zrig et al. (2025) emphasized the role of AM fungi in modulating antioxidant defense systems through Zn-mediated activation of superoxide dismutase and peroxidases, leading to enhanced plant vigor (Zrig et al., 2025). These biochemical changes also contribute to better biomass accumulation and tolerance to environmental stressors. Given the growing concern over micronutrient deficiencies in both crops and human diets, AM symbiosis presents a promising tool for agronomic biofortification, especially when integrated with cropping systems that optimize rhizospheric health and root-microbe interactions.

### 3.10 Iron

The highest Fe content (3.69 mg kg⁻¹) was observed in intercropped AM-treated pumpkins, highlighting the potential of AM fungi in enhancing micronutrient uptake (**Figure 7B**). Iron is vital for photosynthesis and respiration, and its deficiency commonly limits plant performance in calcareous or alkaline soils (Chen and Barak, 1982). Founoune-Mboup et al. (2024) reported that AM fungi improved Fe translocation not only in roots but also in shoots of pearl millet, increasing both yield and nutritional quality (Founoune-Mboup et al., 2024). Similarly, Ren et al. (2023) found that AM inoculation upregulated Fe transporters like NtYSL7, crucial for maintaining Fe homeostasis and preventing chlorosis (Ren et al., 2023). The benefits of intercropping may be amplified by complementary root exudation and soil microbial activity that enhances Fe solubility. This makes the integration of AM fungi in intercropping systems a powerful strategy for addressing both productivity and nutritional deficiencies in crop production.

## 4 Conclusion

This study demonstrates the substantial influence of arbuscular mycorrhizal (AM) fungi and cropping system design on the physiological performance, nutrient acquisition, and oil composition of sunflower and pumpkin plants. AM inoculation significantly improved total oil yield and oleic acid concentration, particularly under intercropping conditions, highlighting its potential to enhance the nutritional quality of oilseeds. Root colonization was most pronounced in AM-treated intercropped pumpkins, emphasizing the compatibility between plant species and AM fungi in diverse cropping environments.

Furthermore, AM fungi markedly increased chlorophyll content, plant height, leaf number, and vegetative biomass, with intercropped AM treatments producing the most robust growth responses. Nutrient profiling revealed that AM-inoculated plants exhibited significantly higher concentrations of phosphorus, potassium, calcium, zinc, and iron, especially in intercropped systems, suggesting improved mineral nutrient uptake efficiency and potential for crop biofortification.

Collectively, these findings underscore the synergistic benefits of combining AM fungi with intercropping practices to boost crop productivity, enhance seed quality, and promote sustainable nutrient management. The integration of AM symbiosis into cropping strategies offers a viable, eco-friendly approach to improve yield and resilience in modern agriculture, particularly under low-input or marginal soil conditions.

## Data Availability

The raw data supporting the conclusions of this article will be made available by the authors, without undue reservation.
